# Supplementary figures and images for: Antibiotic prescription practices in primary care in low- and middle-income countries: A systematic review and meta-analysis
Source: PLoS Med. 2020 Jun 16;17(6):e1003139. doi: 10.1371/journal.pmed.1003139 (PMC7297306; doi:10.1371/journal.pmed.1003139)

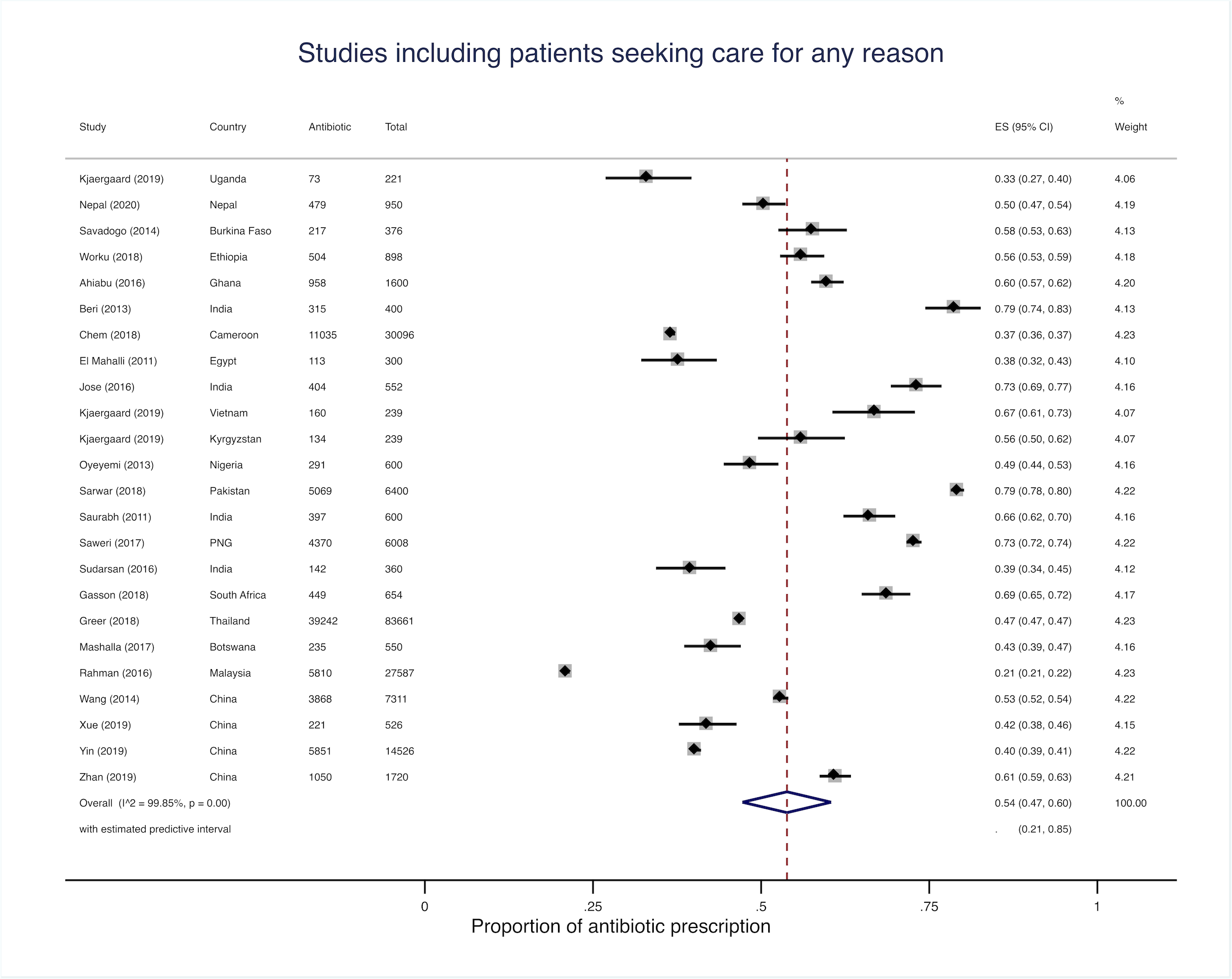

Supplement: S1 Fig — (TIF) [file pmed.1003139.s002.tif]

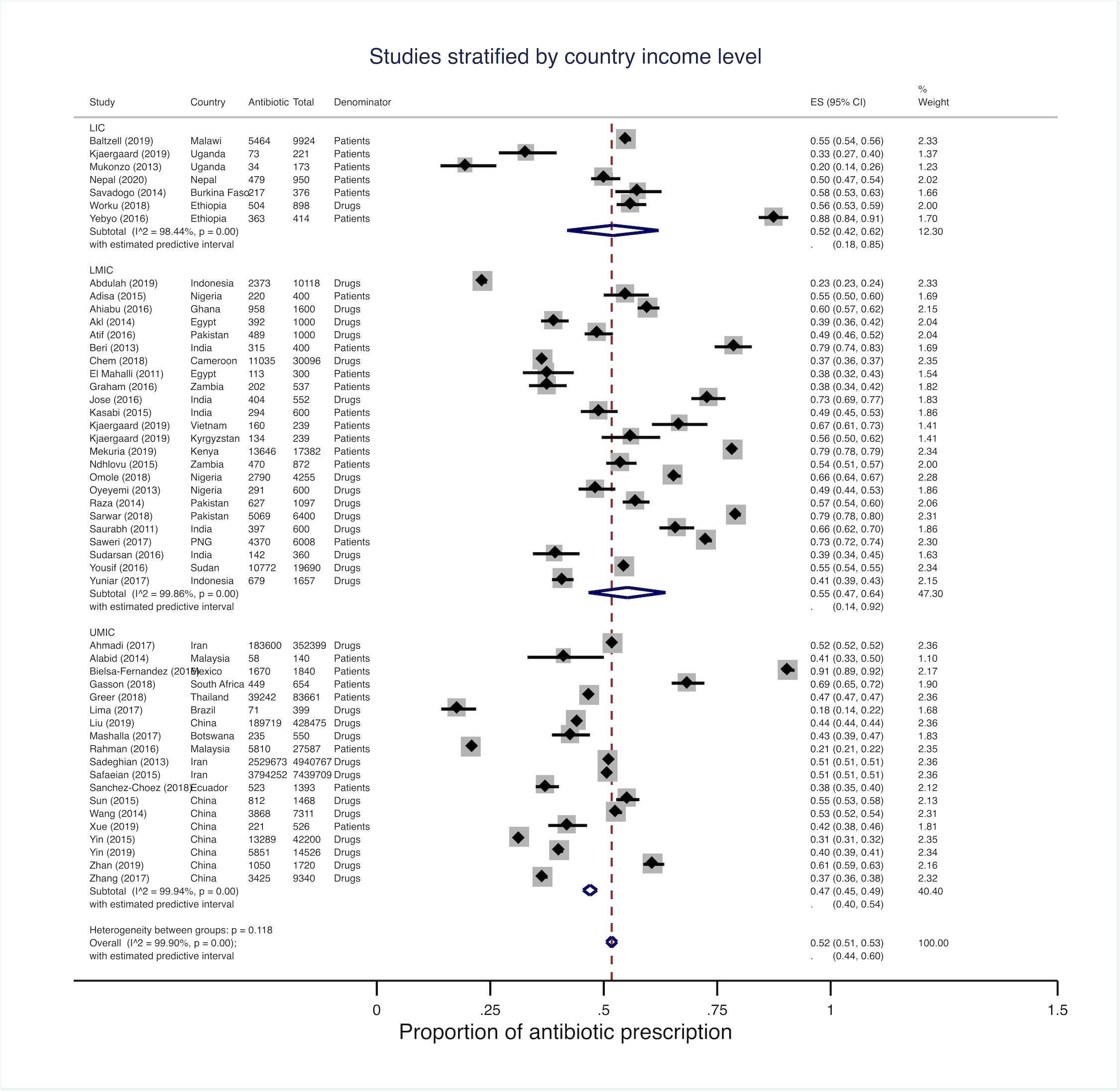

Supplement: S2 Fig — (TIF) [file pmed.1003139.s003.tif]

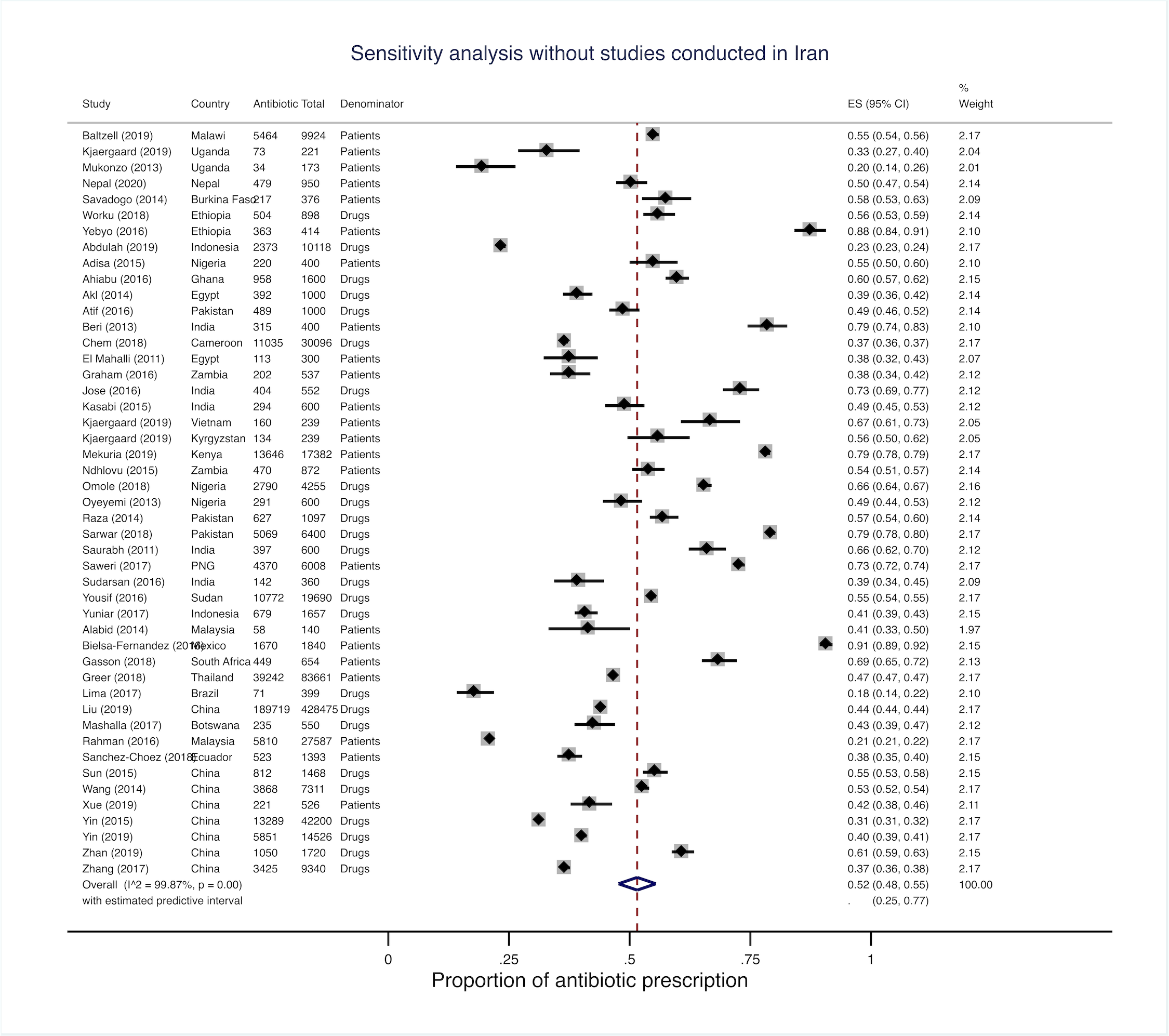

Supplement: S3 Fig — (TIF) [file pmed.1003139.s004.tif]

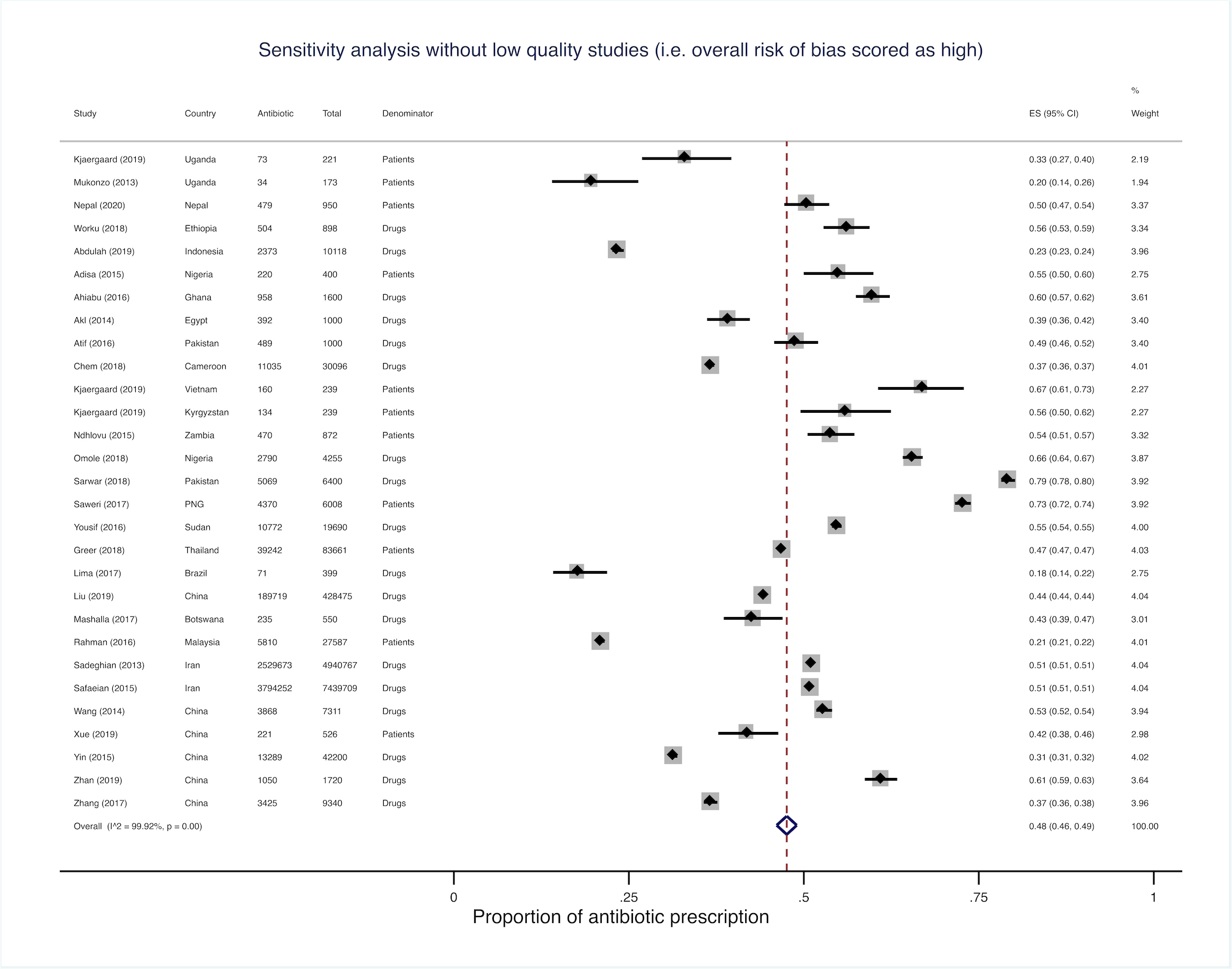

Supplement: S4 Fig — (TIF) [file pmed.1003139.s005.tif]
