# Supplementary material for: Antibiotic prescription practices in primary care in low- and middle-income countries: A systematic review and meta-analysis
Source: PLoS Med. 2020 Jun 16;17(6):e1003139. doi: 10.1371/journal.pmed.1003139 (PMC7297306; doi:10.1371/journal.pmed.1003139)
Supplement: S1 PROSPERO Protocol — (PDF) [file pmed.1003139.s007.pdf]

Systematic review

1. * Review title.

Give the working title of the review, for example the one used for obtaining funding. Ideally the title should state succinctly the interventions or exposures being reviewed and the associated health or social problems. Where appropriate, the title should use the PI(E)COS structure to contain information on the Participants, Intervention (or Exposure) and Comparison groups, the Outcomes to be measured and Study designs to be included.

Antibiotic prescription practices at the primary healthcare level in low- and middle-income countries: a systematic review

2. Original language title.

For reviews in languages other than English, this field should be used to enter the title in the language of the review. This will be displayed together with the English language title.

3. * Anticipated or actual start date.

Give the date when the systematic review commenced, or is expected to commence.

24/01/2019

4. * Anticipated completion date.

Give the date by which the review is expected to be completed.

30/09/2019

5. * Stage of review at time of this submission.

Indicate the stage of progress of the review by ticking the relevant Started and Completed boxes. Additional information may be added in the free text box provided.
Please note: Reviews that have progressed beyond the point of completing data extraction at the time of initial registration are not eligible for inclusion in PROSPERO. Should evidence of incorrect status and/or completion date being supplied at the time of submission come to light, the content of the PROSPERO record will be removed leaving only the title and named contact details and a statement that inaccuracies in the stage of the review date had been identified.
This field should be updated when any amendments are made to a published record and on completion and publication of the review. If this field was pre-populated from the initial screening questions then you are not able to edit it until the record is published.

The review has not yet started: No

Review stage	Started	Completed
Preliminary searches	Yes	No
Piloting of the study selection process	No	No
Formal screening of search results against eligibility criteria	No	No
Data extraction	No	No
Risk of bias (quality) assessment	No	No
Data analysis	No	No

Provide any other relevant information about the stage of the review here (e.g. Funded proposal, protocol not yet finalised).

6. * Named contact.

The named contact acts as the guarantor for the accuracy of the information presented in the register record.

Giorgia Sulis

Email salutation (e.g. "Dr Smith" or "Joanne") for correspondence:

Dr Sulis

7. * Named contact email.

Give the electronic mail address of the named contact.

giorgia.sulis@mail.mcgill.ca

8. Named contact address

Give the full postal address for the named contact.

McGill University, Department of Epidemiology, Biostatistics and Occupational Health, 1020 Pine Avenue W,

H3A 1A2 Montreal, QC, Canada

9. Named contact phone number.

Give the telephone number for the named contact, including international dialling code.

+1 514 659 6320

10. * Organisational affiliation of the review.

Full title of the organisational affiliations for this review and website address if available. This field may be completed as 'None' if the review is not affiliated to any organisation.

McGill University

Organisation web address:

11. * Review team members and their organisational affiliations.

Give the title, first name, last name and the organisational affiliations of each member of the review team. Affiliation refers to groups or organisations to which review team members belong.

Dr Giorgia Sulis. McGill University
Vaidehi Nafade. McGill University
Sumanth Gandra. Washington University School of Medicine in St. Louis
Benjamin Daniels. World Bank
Jishnu Das. World Bank
Madhukar Pai. McGill University

12. * Funding sources/sponsors.

Give details of the individuals, organizations, groups or other legal entities who take responsibility for initiating, managing, sponsoring and/or financing the review. Include any unique identification numbers assigned to the review by the individuals or bodies listed.

None.

13. * Conflicts of interest.

List any conditions that could lead to actual or perceived undue influence on judgements concerning the main topic investigated in the review.

None

14. Collaborators.

Give the name and affiliation of any individuals or organisations who are working on the review but who are not listed as review team members.

15. * Review question.

State the question(s) to be addressed by the review, clearly and precisely. Review questions may be specific or broad. It may be appropriate to break very broad questions down into a series of related more specific questions. Questions may be framed or refined using PI(E)COS where relevant.

The questions this systematic review aims to answer are:

1) What proportion of patients receive an antibiotic prescription at the primary healthcare level in low- and middle-income countries?

and

2) What proportion of such prescriptions is deemed to be appropriate?

The objectives of this study are to:

• Estimate the overall proportion of patients who received any antibiotic prescription, and, if reported, the overall proportion of antibiotic use that was deemed to be unnecessary or incorrect.

• Estimate the proportion of patients who received an antibiotic prescription stratified by antibiotic class.

• Estimate the proportion of patients who received any antibiotic prescription stratified by health condition.

16. * Searches.

Give details of the sources to be searched, search dates (from and to), and any restrictions (e.g. language or publication period). The full search strategy is not required, but may be supplied as a link or attachment.

The search will be performed following the Preferred Reporting Items for Systematic Reviews and Meta-

Analyses (PRISMA) guidelines. We will work with a medical librarian to systematically search the following electronic databases: PubMed/MEDLINE, EMBASE, Cochrane Library, Global Health and the International Pharmaceutical Abstracts.

The search will be based on key terms such as; “antimicrobial” or “antibiotic” or “anti-infective agent” and “primary care”, and it will include relevant studies published from January 1, 2010 through present, without any language restriction.

17. URL to search strategy.

Give a link to a published pdf/word document detailing either the search strategy or an example of a search strategy for a specific database if available (including the keywords that will be used in the search strategies), or upload your search strategy.Do NOT provide links to your search results.

Alternatively, upload your search strategy to CRD in pdf format. Please note that by doing so you are consenting to the file being made publicly accessible.

Do not make this file publicly available until the review is complete

18. * Condition or domain being studied.

Give a short description of the disease, condition or healthcare domain being studied. This could include health and wellbeing outcomes.

Antimicrobial resistance (AMR) is a major public health concern globally. The inappropriate use of antibiotics plays a key role in the development and spread of AMR, and the optimization of antimicrobial use in humans and animals is among the top-five priorities of the Global Action Plan launched by the WHO in 2015 to tackle AMR. According to a recent analysis of drug sales data in 76 countries, global antibiotic consumption increased by 65% between 2000 and 2015. In LMICs, high burden of infectious diseases, the lack of regulations concerning drug prescription and over-the-counter sale of antibiotics, the inadequate training of healthcare professionals on rationale use of medicines, and the limited availability of essential diagnostics that leads to large-scale empirical use of antibiotics are all important factors contributing to the level of antibiotic use. However, limited information is available on the degree and type of antibiotic use in outpatient primary healthcare facilities in such contexts, thus making any intervention to promote the rational use of antibiotics particularly challenging.

19. * Participants/population.

Give summary criteria for the participants or populations being studied by the review. The preferred format includes details of both inclusion and exclusion criteria.

This study will include all individuals, regardless of age, sex or pregnancy status, for which information on antibiotic prescription is available. We have defined adults to be persons 15 years of age or older, and children as those who are less than 15 years old.

20. * Intervention(s), exposure(s).

Give full and clear descriptions or definitions of the nature of the interventions or the exposures to be reviewed.

Antibiotic prescription to patients attending outpatient services at the primary healthcare level in LMICs.

21. * Comparator(s)/control.

Where relevant, give details of the alternatives against which the main subject/topic of the review will be compared (e.g. another intervention or a non-exposed control group). The preferred format includes details of both inclusion and exclusion criteria.

Not applicable.

22. * Types of study to be included.

Give details of the types of study (study designs) eligible for inclusion in the review. If there are no restrictions on the types of study design eligible for inclusion, or certain study types are excluded, this should be stated. The preferred format includes details of both inclusion and exclusion criteria.

We will include studies conducted in LMICs that report the proportion of subjects receiving any antibiotic prescription at the primary healthcare level. Studies eligible for inclusion will be cross-sectional studies, prospective and retrospective cohort studies, randomized controlled trials, reports on programmatic evaluations and time-series analyses. No restriction will be placed on age, sex, or pregnancy status of the study participants

We will exclude qualitative studies, economic analyses, mathematical modelling studies, commentaries and editorials. Reports of antibiotic sales and those concerning direct dispensing of antibiotics by pharmacies without reference to a physician’s prescription will not be considered. Studies conducted solely in an inpatient setting, those that focused on veterinary use of antibiotics, and those focused on special cohorts (e.g. patients with cystic fibrosis or neutropenia or other underlying conditions that may justify an increased empirical use of antibiotics, or patients receiving antibiotics as part of prophylactic regimens), will also be excluded.

23. Context.

Give summary details of the setting and other relevant characteristics which help define the inclusion or exclusion criteria.

24. * Main outcome(s).

Give the pre-specified main (most important) outcomes of the review, including details of how the outcome is defined and measured and when these measurement are made, if these are part of the review inclusion criteria.

Antibiotic use will be estimated as the ratio of the number of individuals receiving at least one antibiotic prescription to the number of persons attending a given outpatient clinic within a specified time period.

Timing and effect measures

25. * Additional outcome(s).

List the pre-specified additional outcomes of the review, with a similar level of detail to that required for main outcomes. Where there are no additional outcomes please state ‘None’ or ‘Not applicable’ as appropriate to the review

None.

Timing and effect measures

26. * Data extraction (selection and coding).

Give the procedure for selecting studies for the review and extracting data, including the number of researchers involved and how discrepancies will be resolved. List the data to be extracted.

Search results will be imported into a reference management database, and duplicate citations will be removed. Two reviewers will independently review the title and abstract of all studies identified by the search. To determine whether each study meets inclusion and exclusion criteria as described above, the same two reviewers will independently review the full text of all selected studies. Any queries or disagreements will be resolved with a third reviewer, after which a final list of articles and literature will be produced as a consensus of all three reviewers.

An electronic data extraction form will be created and pilot-tested on five randomly selected studies. Once the form has been finalized, two independent reviewers will extract data on study methodology, quality and predefined outcomes from the final list of included studies. Disagreements or queries will be resolved between the review authors; if no agreement can be reached, a third author will mediate and decide on the issue. In the case that data are not reported at the level required for each analysis, we will contact authors directly by email.

The following main data items will be collected (this is a non-exhaustive list):

- Study location
- Outcome(s) definition
- Source of information concerning antibiotic prescription (e.g. patients' records, exit interviews, clinic database, registers, prescription audits)
- Types of healthcare providers involved (i.e. physicians, nurses, others)
- Healthcare sector (i.e. private or public facility or informal sector)
- Types of antibiotics used (if available)
- Patients' characteristics (age, sex, medical conditions for which they were seeking care)
- Number of subjects who attended the facility over the study period
- Number of subjects who were prescribed a medication
- Number of subjects who were prescribed one or more antibiotics.
- Percentage of antibiotics that were deemed to be unnecessary or incorrect and methodology used to make this judgement.

27. * Risk of bias (quality) assessment.

State whether and how risk of bias will be assessed (including the number of researchers involved and how discrepancies will be resolved), how the quality of individual studies will be assessed, and whether and how this will influence the planned synthesis.

The two reviewers responsible for data extraction will independently evaluate the risk of bias and internal validity of each included study, using an adapted version of a tool developed by Hoy D. and colleagues for prevalence studies. Reviewers will evaluate each study to ensure that its design and conduct did not compromise the integrity of the results, irrespective of the specific study design utilized. As with study selection, any disagreements or queries with regards to methodological quality will be resolved by a third reviewer. Findings from this assessment will be recorded within the data extraction form.

28. * Strategy for data synthesis.

Give the planned general approach to synthesis, e.g. whether aggregate or individual participant data will be used and whether a quantitative or narrative (descriptive) synthesis is planned. It is acceptable to state that a quantitative synthesis will be used if the included studies are sufficiently homogenous.

Utilizing the data collected through the systematic review, meta-analyses will be conducted if heterogeneity is not a concern.

For each study, we will report the proportion (and 95% confidence interval) of patients receiving at least one antibiotic prescription, as described above. Heterogeneity will be assessed using the I^2 statistic.

As we anticipate substantial between-studies heterogeneity, the proportions of antibiotic prescriptions will be pooled using random effects meta-analysis, and subgroup analyses will be used to identify sources of heterogeneity.

29. * Analysis of subgroups or subsets.

Give details of any plans for the separate presentation, exploration or analysis of different types of participants (e.g. by age, disease status, ethnicity, socioeconomic status, presence or absence or co-morbidities); different types of intervention (e.g. drug dose, presence or absence of particular components of intervention); different settings (e.g. country, acute or primary care sector, professional or family care); or different types of study (e.g. randomised or non-randomised).

Random-effects weighted proportions will be assessed for the following subgroups: major health conditions (e.g. febrile illness, respiratory syndrome, gastrointestinal syndrome, genitourinary syndrome, etc), age (adults vs. children), males vs. females, empirical vs. diagnosis-driven prescription, provider-type (physician vs. non-physician), healthcare sector (public vs. private).

30. * Type and method of review.

Select the type of review and the review method from the lists below. Select the health area(s) of interest for your review.

Type of review

Cost effectiveness
No

Diagnostic
No

Epidemiologic
No

Individual patient data (IPD) meta-analysis
No

Intervention

No

Meta-analysis
No

Methodology
No

Narrative synthesis
No

Network meta-analysis
No

Pre-clinical
No

Prevention
No

Prognostic
No

Prospective meta-analysis (PMA)
No

Review of reviews
No

Service delivery
No

Synthesis of qualitative studies
No

Systematic review
Yes

Other
No

Health area of the review

Alcohol/substance misuse/abuse
No

Blood and immune system
No

Cancer
No

Cardiovascular
No

Care of the elderly
No

Child health
No

Complementary therapies
No

Crime and justice
No

Dental
No

Digestive system
No

Ear, nose and throat

No

Education
No

Endocrine and metabolic disorders
No

Eye disorders
No

General interest
No

Genetics
No

Health inequalities/health equity
No

Infections and infestations
Yes

International development
No

Mental health and behavioural conditions
No

Musculoskeletal
No

Neurological
No

Nursing
No

Obstetrics and gynaecology
No

Oral health
No

Palliative care
No

Perioperative care
No

Physiotherapy
No

Pregnancy and childbirth
No

Public health (including social determinants of health)
Yes

Rehabilitation
No

Respiratory disorders
No

Service delivery
Yes

Skin disorders
No

Social care
No

Surgery
No

Tropical Medicine
No

Urological
No

Wounds, injuries and accidents
No

Violence and abuse
No

31. Language.

Select each language individually to add it to the list below, use the bin icon to remove any added in error.
English

There is not an English language summary

32. Country.

Select the country in which the review is being carried out from the drop down list. For multi-national collaborations select all the countries involved.

Canada

33. Other registration details.

Give the name of any organisation where the systematic review title or protocol is registered (such as with The Campbell Collaboration, or The Joanna Briggs Institute) together with any unique identification number assigned. (N.B. Registration details for Cochrane protocols will be automatically entered). If extracted data will be stored and made available through a repository such as the Systematic Review Data Repository (SRDR), details and a link should be included here. If none, leave blank.

34. Reference and/or URL for published protocol.

Give the citation and link for the published protocol, if there is one

Give the link to the published protocol.

Alternatively, upload your published protocol to CRD in pdf format. Please note that by doing so you are consenting to the file being made publicly accessible.

No I do not make this file publicly available until the review is complete

Please note that the information required in the PROSPERO registration form must be completed in full even if access to a protocol is given.

35. Dissemination plans.

Give brief details of plans for communicating essential messages from the review to the appropriate audiences.

Results from this review will be submitted for publication to a peer-reviewed journal and will also be included in a PhD thesis at McGill University (GS).

Do you intend to publish the review on completion?

Yes

36. Keywords.

Give words or phrases that best describe the review. Separate keywords with a semicolon or new line. Keywords will help users find the review in the Register (the words do not appear in the public record but are included in searches). Be as specific and precise as possible. Avoid acronyms and abbreviations unless these are in wide use.

37. Details of any existing review of the same topic by the same authors.

Give details of earlier versions of the systematic review if an update of an existing review is being registered, including full bibliographic reference if possible.

38. * Current review status.

Review status should be updated when the review is completed and when it is published. For newregistrations the review must be Ongoing.
Please provide anticipated publication date

Review_Ongoing

39. Any additional information.

Provide any other information the review team feel is relevant to the registration of the review.

40. Details of final report/publication(s).

This field should be left empty until details of the completed review are available.

Give the link to the published review.
