# Supplementary material for: Antibiotic prescription practices in primary care in low- and middle-income countries: A systematic review and meta-analysis
Source: PLoS Med. 2020 Jun 16;17(6):e1003139. doi: 10.1371/journal.pmed.1003139 (PMC7297306; doi:10.1371/journal.pmed.1003139)
Supplement: S1 Table — (DOCX) [file pmed.1003139.s008.docx]

**S1 Table:** World Bank criteria for the definition of countries’ income level 2010-2018.

| Country income | 2010 | 2011 | 2012 | 2013 | 2014 | 2015 | 2016 | 2017 | 2018 |
| --- | --- | --- | --- | --- | --- | --- | --- | --- | --- |
| **Low** | <= 1,005 | <= 1,025 | <= 1,035 | <= 1,045 | <= 1,045 | <= 1,025 | <= 1,005 | <= 995 | <= 1,025 |
| **Lower-middle** | 1,006-3,975 | 1,026-4,035 | 1,036-4,085 | 1,046-4,125 | 1,046-4,125 | 1,026-4,035 | 1,006-3,955 | 996-3,895 | 1,026-3,995 |
| **Upper-middle** | 3,976-12,275 | 4,036-12,475 | 4,086-12,615 | 4,126-12,745 | 4,126-12,735 | 4,036-12,475 | 3,956-12,235 | 3,896-12,055 | 3,996-12,375 |
| **High** | > 12,275 | > 12,475 | > 12,615 | > 12,745 | > 12,735 | > 12,475 | > 12,235 | > 12,055 | > 12,375 |

Note: Country income categories are defined as gross national income (GNI) per capita in US dollars in accordance to World Bank criteria for each fiscal year (available at: <https://datahelpdesk.worldbank.org/knowledgebase/articles/906519-world-bank-country-and-lending-groups>).
