# Supplementary material for: Antibiotic prescription practices in primary care in low- and middle-income countries: A systematic review and meta-analysis
Source: PLoS Med. 2020 Jun 16;17(6):e1003139. doi: 10.1371/journal.pmed.1003139 (PMC7297306; doi:10.1371/journal.pmed.1003139)
Supplement: S2 Table — (DOCX) [file pmed.1003139.s009.docx]

**S2 Table:** Risk of bias assessment tool [adapted from Hoy et al (2012)]

| **Risk of bias item** | **Criteria for answers** |
| --- | --- |
| **External validity** | |
| 1. Was the study’s target population **a close representation** of the population of interest in relation to relevant variables, e.g. age, sex, occupation, health status or other? | - **Yes (LOW RISK):** The study’s target population was a close representation of the national population. - **No (HIGH RISK):** The study’s target population was clearly NOT representative of the national population. |
| 2. Was the sampling frame a **true or close representation** of the target population? | - **Yes (LOW RISK):** The sampling frame was a true or close representation of the target population. - **No (HIGH RISK):** The sampling frame was NOT a true or close representation of the target population. |
| 3. Was some form of **random selection** used to select the sample, OR, was a census undertaken? | - **Yes (LOW RISK):** A census was undertaken, OR, some form of random selection was used to select the sample (e.g. simple random sampling, stratified random sampling, cluster sampling, systematic sampling). - **No (HIGH RISK):** A census was NOT undertaken, AND some form of random selection was NOT used to select the sample. |
| 4. Did the study avoid inappropriate exclusions? | - **No (LOW RISK)** - **Yes (HIGH RISK)** |
| **Internal validity** | |
| 5. Was an acceptable case definition used in the study? | - **Yes (LOW RISK):** An acceptable case definition was used. - **No (HIGH RISK):** An acceptable case definition was NOT used. |
| 6. Is the study method for measuring drug prescription shown to have **reliability and validity (if necessary)**? i.e. is there an opportunity for misclassification | - **Yes (LOW RISK):** The method is shown to have minimal misclassification potential - **No (HIGH RISK):** The method is NOT shown to have minimal misclassification potential |
| 7. Was the **same mode of data collection** used for all subjects? | - **Yes (LOW RISK):** The same mode of data collection was used for all subjects. - **No (HIGH RISK):** The same mode of data collection was NOT used for all subjects. |
| 8. Were the **numerator(s) and denominator(s)** for the parameter of interest appropriate? | - **Yes (LOW RISK):** The paper presented appropriate numerator(s) AND denominator(s) for the parameter of interest. - **No (HIGH RISK):** The paper did present numerator(s) AND denominator(s) for the parameter of interest but one or more of these were inappropriate. |
| **Summary item on the overall risk of study bias** | |
| - **LOW RISK OF BIAS:** Further research is very unlikely to change our confidence in the estimate. - **MODERATE RISK OF BIAS:** Further research is likely to have an important impact on our confidence in the estimate and may change the estimate. - **HIGH RISK OF BIAS:** Further research is very likely to have an important impact on our confidence in the estimate and is likely to change the estimate. | |
