# Supplementary material for: Antibiotic prescription practices in primary care in low- and middle-income countries: A systematic review and meta-analysis
Source: PLoS Med. 2020 Jun 16;17(6):e1003139. doi: 10.1371/journal.pmed.1003139 (PMC7297306; doi:10.1371/journal.pmed.1003139)
Supplement: S3 Table — (DOCX) [file pmed.1003139.s010.docx]

**S3 Table:** Risk of bias assessment of all studies included in final synthesis.

| **Study** | **Representative of the target population** | **Appropriate sampling frame used** | **Random selection or census used** | **Avoidance of inappropriate exclusions** | **Appropriate case definition** | **Reliability & validity of data collection method** | **Same mode of data collection for all subjects** | **Appropriate numerators and denominators** | **Overall risk of bias** |
| --- | --- | --- | --- | --- | --- | --- | --- | --- | --- |
| Abdulah et al, Drug Healthc Patient Saf (2019) | HR | LR | LR | LR | HR | LR | LR | LR | **MODERATE** |
| Xue et, J Antimicrob Chemother (2019) | LR | LR | HR | LR | LR | LR | LR | LR | **MODERATE** |
| Sarwar et al, BMC Infect Dis (2018) | LR | LR | LR | LR | HR | LR | LR | LR | **MODERATE** |
| Greer et al, BMJ Open (2018) | LR | LR | LR | LR | LR | LR | LR | LR | **LOW** |
| Sanchez-Choez et al, BMC Pharmacol Toxicol (2018) | HR | HR | HR | LR | LR | LR | LR | LR | **HIGH** |
| Worku et al, Interdiscip Prospect Infect Dis (2018) | LR | LR | LR | LR | LR | LR | LR | LR | **LOW** |
| Gasson et al, S Afr Med J (2018) | HR | HR | LR | HR | LR | LR | LR | LR | **HIGH** |
| Chem et al, PLoS ONE (2018) | HR | LR | LR | LR | LR | LR | LR | LR | **MODERATE** |
| Ahmadi et al, BMC Public Health (2017) | HR | LR | HR | LR | HR | LR | LR | LR | **HIGH** |
| Mashalla et al, Int J Clin Pract (2017) | HR | LR | LR | LR | LR | LR | LR | LR | **MODERATE** |
| Lima et al, Int J Clin Pharm (2017) | LR | LR | LR | LR | LR | LR | LR | LR | **LOW** |
| Zhang et al, Glob Health Action (2017) | LR | LR | LR | LR | LR | LR | LR | LR | **LOW** |
| Jose et al, J Clin Diagn Res (2016) | HR | HR | HR | LR | LR | LR | LR | LR | **HIGH** |
| Atif et al, BMC Health Serv Res (2016) | LR | LR | LR | LR | LR | LR | LR | LR | **LOW** |
| Yousif et al, Drugs Real World Outcomes (2016) | LR | LR | LR | LR | HR | LR | LR | LR | **MODERATE** |
| Graham et al, BMC Public Health (2016) | HR | HR | HR | LR | LR | LR | LR | LR | **HIGH** |
| Rahman et al, BMC Infect Dis (2016) | LR | LR | LR | LR | LR | LR | LR | LR | **LOW** |
| Adisa et al, Afr Health Sci (2015) | LR | LR | HR | LR | LR | LR | LR | LR | **MODERATE** |
| Yebyo et al, NPJ Prim Care Resp Med (2016) | HR | HR | HR | LR | LR | LR | LR | LR | **HIGH** |
| Yin et al, Med Care (2015) | LR | LR | LR | LR | LR | LR | LR | LR | **LOW** |
| Ndhlovu et al, Trop Med Int Health (2015) | LR | LR | LR | LR | LR | LR | HR | LR | **LOW** |
| Ahiabu et al, Health Policy Plan (2016) | LR | LR | LR | LR | LR | LR | LR | LR | **LOW** |
| Sun et al, BMC Pharmacol Toxicol (2015) | LR | HR | HR | LR | LR | LR | LR | LR | **HIGH** |
| Safaeian, et al, Int J Prev Med (2015) | LR | LR | LR | LR | LR | LR | LR | LR | **LOW** |
| Wang et al, JAMA Intern Med (2014) | LR | LR | LR | LR | LR | LR | LR | LR | **LOW** |
| Raza et al, Pak J Med Sci (2014) | HR | HR | HR | LR | HR | LR | LR | LR | **HIGH** |
| Alabid et al, J Clin Diagn Res (2014) | HR | HR | HR | LR | LR | LR | LR | LR | **HIGH** |
| Sadeghian et al, Iran J Pharm Res (2013) | LR | LR | LR | LR | LR | LR | LR | LR | **LOW** |
| Oyeyemi et al, West Afr J Med (2013) | HR | HR | LR | LR | LR | LR | LR | LR | **HIGH** |
| Beri et al, J Clin Diagn Res (2013) | HR | HR | HR | LR | LR | HR | LR | LR | **HIGH** |
| El Mahalli et al, J Fam Community Med (2011) | HR | HR | LR | LR | LR | LR | LR | LR | **HIGH** |
| Kasabi et al, Indian J Med Res (2015) | HR | HR | HR | LR | LR | LR | LR | LR | **HIGH** |
| Omole et al, Int J Med Health Dev (2018) | LR | LR | LR | LR | HR | LR | LR | LR | **MODERATE** |
| Savadogo et al, Health (2014) | HR | HR | HR | LR | LR | LR | LR | LR | **HIGH** |
| Saweri et al, BMC Health Serv Res (2017) | LR | LR | LR | LR | LR | LR | HR | LR | **LOW** |
| Sudarsan et al, Int J Med Public Health (2016) | HR | HR | HR | LR | LR | LR | LR | LR | **HIGH** |
| Akl et al, J Taibah Univ Med Sci (2014) | LR | LR | LR | LR | HR | LR | LR | LR | **MODERATE** |
| Bielsa-Fernandez et al, Atención Familiar (2016) | HR | HR | HR | LR | LR | HR | LR | LR | **HIGH** |
| Mukonzo et al, J Multidiscip Healthc (2013) | LR | LR | LR | LR | HR | LR | HR | LR | **MODERATE** |
| Saurabh et al, Asian J Pharmacy Clin Res (2011) | HR | HR | HR | LR | LR | LR | LR | LR | **HIGH** |
| Yuniar et al, Jurnal Kefarmasian Indonesia (2017) | HR | HR | HR | LR | HR | LR | LR | LR | **HIGH** |
| Baltzell et al, Rural Remote Health (2019) | LR | HR | HR | LR | LR | LR | LR | LR | **HIGH** |
| Kjaergaard et al, PloS One (2019) | HR | LR | LR | LR | LR | LR | LR | LR | **MODERATE** |
| Liu et al, Antimicrob Resist Infect Control (2019) | LR | LR | LR | LR | LR | LR | LR | LR | **LOW** |
| Mekuria et al, PloS One (2019) | HR | HR | HR | LR | LR | LR | LR | LR | **HIGH** |
| Nepal et al, J Infect Dev Ctries (2020) | LR | LR | LR | LR | LR | LR | LR | LR | **LOW** |
| Yin et al, Trans R Soc Trop Med Hyg (2019) | HR | HR | HR | LR | LR | LR | LR | LR | **HIGH** |
| Zhan et al, J Glob Antimicrob Resist (2019) | LR | LR | LR | LR | LR | LR | LR | LR | **LOW** |

Abbreviations: HR = high risk; LR = low risk
