# Supplementary material for: Antibiotic prescription practices in primary care in low- and middle-income countries: A systematic review and meta-analysis
Source: PLoS Med. 2020 Jun 16;17(6):e1003139. doi: 10.1371/journal.pmed.1003139 (PMC7297306; doi:10.1371/journal.pmed.1003139)
Supplement: S4 Table — (DOCX) [file pmed.1003139.s011.docx]

**S4 Table:** Results of meta-regression analysis

| Predictor | OR | 95% CI |
| --- | --- | --- |
| LMIC | **1.06** | **0.91 – 1.24** |
| UMIC | **0.92** | **0.78 – 1.07** |
| Urban areas only | **0.91** | **0.79 – 1.04** |
| Both urban and rural areas | **0.92** | **0.80 – 1.06** |
| Public sector | **0.88** | **0.73 – 1.07** |
| Source of data | **1.03** | **0.85 – 1.24** |

Notes: The inclusion of calendar time (i.e. study start year) had no effect on the model’s performance. Similar considerations apply to overall risk-of-bias scores.

Abbreviations: CI = Confidence interval; LMIC = Lower-middle income country; OR = Odds ratio; UMIC = Upper-middle income country.
