# Supplementary material for: Antibiotic prescription practices in primary care in low- and middle-income countries: A systematic review and meta-analysis
Source: PLoS Med. 2020 Jun 16;17(6):e1003139. doi: 10.1371/journal.pmed.1003139 (PMC7297306; doi:10.1371/journal.pmed.1003139)
Supplement: S2 Text — (DOCX) [file pmed.1003139.s013.docx]

**S2 Text:** Selection process

The study screening was performed through a three-step process. First, three authors (GS, PA and VN) conducted a title-based screening, assessing one third of all citations each. At this stage, we adopted a highly conservative approach, only excluding records whose title clearly referred to high-income settings, hospitals providing services other than primary care, animal studies, conditions or approaches that were totally unrelated to our study question (e.g. non-communicable diseases, HIV, diagnostic accuracy studies). All records that were discarded by one of the three reviewers were double-checked by another reviewer and retained for further assessment in the event of disagreement. The interrater agreement during this phase of the screening process was excellent (>95%).

Second, the same three authors as above screened all abstracts that were selected during step 1. The following exclusion criteria were considered to make a decision:

- Conference proceedings and abstracts;
- Commentaries or editorials;
- Reviews;
- Mathematical modelling studies;
- Economic analyses;
- Qualitative studies;
- Studies conducted only in an inpatient setting;
- Studies focused on veterinary or agricultural use of antibiotics;
- Studies focused exclusively on specials cohorts such as (i) patients with cystic fibrosis or neutropenia or other underlying conditions that may justify an increased empirical use of antibiotics, or (ii) patients receiving antibiotics as part of prophylactic regimens (e.g. cotrimoxazole preventive therapy provided to HIV-infected individuals).

If any of the aforementioned criteria could not be ruled out from the abstract only, the publication was retained for full-text evaluation. All abstracts that were selected by each reviewer were jointly discussed to reach consensus about inclusion or exclusion.

Third, two authors (GS and PA) applied the same criteria as above to perform the full-text screening. Publications from predatory journals defined in accordance with Beall’s list (<https://beallslist.net/standalone-journals/>) were excluded. To validate exclusion/inclusion based on the Beall’s list, we also used the item checklist suggested in the Think-Check tool (<https://thinkchecksubmit.org/check/>). At this stage of the screening process, the reviewers also devoted attention to the level of care involved in the studies being examined and only those conducted in primary care were selected. In case of uncertainties regarding the level of care, or if multiple tiers of the health system were evaluated, the study authors were contacted for clarifications and/or to request additional information including disaggregated data where available.

The overall percent agreement between reviewers regarding allocation of full-text publications to one of three categories (“included”, “excluded”, “authors to be contacted”) was 69.5%. The Randolph’s free-marginal kappa statistic was 54%, suggesting an intermediate to good interrater agreement.

Any discrepancies were discussed until consensus was reached. A senior authors (SG) was consulted to finalize the decisions on inclusion and exclusion of individual studies into final analyses.

**Data extracted from each study included in final synthesis:**

1. Bibliographic information
2. Study information:

- Study design;
- Study period;
- Study site: geographic region as per WHO classification, country, income level as per World Bank classification, healthcare sector, type of health facility and providers, facility location (urban or rural area);
- Sampling strategy;
- Source of data (e.g. medical records, drug prescription audits, patient exit interviews, provider questionnaires, direct observation, other);
- Methods used to assess the appropriateness of antibiotic prescriptions (if any).

1. Population details:
   - Age and sex demographics;
   - HIV status;
   - Reason for seeking care;
2. Antibiotic prescription information:
   - Number of patients evaluated;
   - Number of patients receiving a drug prescription;
   - If available, overall as well as by healthcare sector (public/private), health facility location (urban/rural), age group (adults/children), sex (males/females) and clinical condition (acute respiratory illness, diarrhea/gastroenteritis, genitourinary syndrome, fever):
     1. Number of patients receiving one or more antibiotics;
     2. Number of inappropriate antibiotic prescriptions;
     3. Number of antibiotics prescriptions belonging to each of the three categories of the WHO AWaRe classification (<https://adoptaware.org>).
