# Supplementary material for: Antibiotic prescription practices in primary care in low- and middle-income countries: A systematic review and meta-analysis
Source: PLoS Med. 2020 Jun 16;17(6):e1003139. doi: 10.1371/journal.pmed.1003139 (PMC7297306; doi:10.1371/journal.pmed.1003139)
Supplement: Attachment — Submitted filename: Response to reviewers.docx [file pmed.1003139.s014.docx]

Dr Adya Misra, PhD

Senior Editor

PLOS Medicine

Dear Dr Misra

Thank you very much for the kind consideration you gave to our manuscript entitled “*Antibiotic prescription practices in primary care in low- and middle-income countries: a systematic review and meta-analysis*” and for giving us the opportunity to revise it. We have worked hard at the revision, including doing a new PubMed search and inclusion of new papers.

Our point-by-point reply to the reviewer’s comments is below. A revised version of our manuscript has also been submitted. Please note that both the PRISMA checklist is provided as supplementary table S1, and the PRISMA diagram is provided as Figure 1.

Hoping this version will meet your approval, we look forward to receiving your valuable feedback.

Kindest regards,

Prof. Madhukar Pai

Corresponding author

**Requests from the editors:**

1. Abstract- the last sentence of the methods and findings section should include a limitation of your study design.

**Thanks for your suggestion. Limitations have been added to the abstract.**

2. Abstract- perhaps structure abstract according to PRISMA guidelines.

**The abstract is now structured according to PRISMA guidelines with relevant sub-headings.**

3. Literature search- Please update this to current date, as we are coming up to a year since the last search.

**Because a rigorous screening and data extraction process requires a long time, we updated our search using PubMed only instead of four different data sources as done in the initial search (which was conducted less than 12 months ago). The manuscript has been modified accordingly.**

4. At this stage, we ask that you include a short, non-technical Author Summary of your research to make findings accessible to a wide audience that includes both scientists and non-scientists. The Author Summary should immediately follow the Abstract in your revised manuscript. This text is subject to editorial change and should be distinct from the scientific abstract. Please see our author guidelines for more information: <https://journals.plos.org/plosmedicine/s/revising-your-manuscript#loc-author-summary>.

**The Author Summary has been added as requested.**

5. Reviewers have noted the definition of primary care must be defined for clarity, please include this in the introduction and methods section.

**Thank you for pointing this out. We have included a broad definition of “primary care” in the introduction (Lines 137-139), whereas a more detailed description is given in the methods section (Lines 165-168).**

6. PRISMA checklist- We ask that you include a sentence in your methods section that the study followed PRISMA guidelines and that a completed checklist has been provided as SI file xx. In addition, please use paragraphs and sections instead of page numbers as they are likely to change.

**We had already mentioned at the beginning of the methods section that PRISMA guidelines were followed. We have now included a reference to the PRISMA checklist (now S1 Table) provided in the supplementary material (Line 148). Paragraphs and sections were included in the checklist as requested.**

7. Methods- if all study types were included in the search this sentence is confusing “We performed a systematic review of cross-sectional studies”. Please correct and clarify as needed.

**Thank you for highlighting this issue. Our focus was on cross-sectional studies, and all those included in final analyses are cross-sectional. However, we also evaluated other observational studies and randomized trials and searched for results of preliminary field assessments that might have been carried out in preparation for the core study. For instance, it is possible that before conducting an RCT to assess the impact of a given diagnostic tool, a background assessment of routine care practices was performed by researchers, potentially including information on antibiotic prescriptions. We have rephrased the sentence to clarify this point (Lines 153-156).**

8. Please provide a rationale for excluding studies from “predatory” journals, especially as lists of predatory publishers have been deemed controversial and biased.

**Thank you for raising this point. Predatory journals do not involve a peer-review process and do not check articles for methodological quality and legitimacy (even from an ethical standpoint). Such publications do not meet the minimum standards of scientific transparency, so we strongly believe that it is our duty of researchers to prevent them from thriving further. We feel that including these papers in our analyses would be an expression of endorsement. We are aware that currently available lists of predatory journals and publishers are controversial and possibly incomplete. Our reference was the Beall’s list, which is deemed to be the most accurate compilation. We also used the Think-Check tool (**[**https://thinkchecksubmit.org/check/**](https://thinkchecksubmit.org/check/)**) to validate our inclusion/exclusion decision. Notably, only 5 papers were excluded because published in predatory journals, thus likely affecting our findings only marginally.**

9. Methods- please provide a brief summary of how antibiotic prescriptions were deemed to be inappropriate, providing references to tools/checklists as needed.

**Thanks for your suggestion. We did not make any judgement on appropriateness of prescription in individual studies because clinical details were insufficient to do so. This is one of the limitation of studies based on medical records abstraction or drug prescription audits. We have included some clarifications in the manuscript to underline that we only reported proportions of inappropriate antibiotic prescriptions as defined and reported by the study authors who generally made their judgement according to national and/or international guidelines (Lines 170-171 and Lines 330-332).**

10. The conclusions need to be toned down, since the reliability of the pooled estimates is low owing to high heterogeneity between included studies.

**We have reworked the conclusions as requested (Lines 421-439).**

**Comments from the reviewers:**

**Reviewer #1:**

This study reviews and meta-analyses antibiotic prescription practices in primary care in low- and middle-income countries. While it is an interesting topic, I do think the manuscript should be improved before it would be acceptable for publication.

1. Abstract. It is unclear why the authors focus on the prevalence of antibiotic prescriptions. Prevalence gives the proportion of a population who have a specific characteristic in a given time period, e.g. the proportion of the population that is using antibiotics at a given day. It would be more interesting to look at the incidence of antibiotic prescriptions or e.g. of those presenting with symptom x, what proportion of patients gets an antibiotic.

**Thank you for bringing up this issue. Although time series analyses do exist, most studies that seek to evaluate prescription data make of use medical records or cross-sectional drug prescription audits that provide only prevalence estimates, i.e. a snapshot of what is going on at a given point in time. In this this, we only found studies that reported this type of measure. Importantly, although the design is often defined as “cohort study” by research authors, thus suggesting observation over time, all studies were actually cross-sectional. We agree with the reviewer that incidence measures and time trends would be very interesting, and this is something that needs to be considered to improve future research on this issue. Yet, we believe that prevalence estimates are also important and, if taken from an appropriate sample, provide useful information on the magnitude of antibiotic prescription.**

**However, we did try to look for evidence of differences over time and could not identify any apparent time trends based on available data. Three quarters of the studies were conducted in 2014 or earlier and often published years later. The reported proportion of patients receiving antibiotics does not show any specific trend over time, and similar considerations can be done after stratifying for the overall risk of study bias. Including calendar time in meta-regression models did not yield any improvements in the model performance.**

2. I don't understand why the authors try to model a pooled estimate across all years and countries. Surely there are strong trends among prescribing trends in LMIC (which probably also differ per region), as also highlighted by the authors in the introduction. Thus the pooled estimate is impossible to interpret and highly dependent on the number of studies and samples sizes in different periods.

**Thanks for your comment. As we mention in the paper with regards to calendar time, we did try to look at possible trends over time using the study publication year and the year in which the study was conducted, but we could not observe any particular pattern. On the other hand, stratifying by country is impossible given that we retrieved 48 studies conducted across 27 different countries. We are well aware that pooled estimates have to be cautiously interpreted. Yet, we believe that these findings are still interesting. About three quarter of the studies included in our final synthesis reported an average proportion of antibiotic prescribing above 40%, and in most cases this proportion ranged between 40 and 60%, thus showing an overall alarming picture in spite of heterogeneity issues. We have made some changes in the results section and forest plots in order to better account for differences in denominators being used in different studies. We also modified our conclusions as requested by the editors.**

3. Abstract. The proportion of prescriptions ranged from 8 to 100%. Surely this 100% is not correct, a number that is obtained using questionable methods. Given this, please also add a comment on the quality of the included studies.

**Thank you for your comment. As explained in the results section of our paper, the estimate of 100% incorrect antibiotic prescription is actually correct because it comes from studies that used simulated patients (SPs). These patients portrayed clinical conditions like common cold for which antibiotics are never recommended, which resulted in 100% of antibiotics being inappropriately prescribed. This is exactly why the SP methodology provides a unique opportunity to better understand prescription practices.**

4. Introduction. 'An estimated 10 million deaths per year are expected to….' No, they are not expected to die, there are many problems with the report that came up with this number. See, for example <https://www.ncbi.nlm.nih.gov/pmc/articles/PMC5127510/>

**Thanks a lot for bringing this to our attention. We have modified the text accordingly (Lines 113-115).**

5. Introduction. 'The high level of antibiotic consumption in LMICS…' Is it hight, or is it just coming closer to HIC?

**We have modified the text to clarify that the level of consumption in LMICs is getting closer to that observed in HICs (Lines 120-124).**

6. 'Most studies investigating the magnitude and determinants of antibiotic use have been focused on high-income countries (HIC) and particularly on hospital settings.' While the first claim is true, there are many studies estimating the magnitude and determinants of (inappropriate) antibiotic use in primary care/outpatient setting, e.g.

<https://www.bmj.com/content/364/bmj.l440.full>

<https://academic.oup.com/jac/article/73/suppl_2/ii36/4841818>

<https://www.sciencedirect.com/science/article/pii/S2589537018300531>

<https://academic.oup.com/jac/article/73/suppl_2/ii27/4841819>

<https://www.bmj.com/content/362/bmj.k3155>

<https://www.tandfonline.com/doi/full/10.1080/02813432.2017.1288680>

In fact, in the UK we probably have a better understanding of prescribing practice than in the hospital setting. Therefore, the sentence of paucity of information of antibiotic use in outpatient primary healthcare facilities, only applies potentially to LIMC.

**We agree with the reviewer that our statement was confusing. We have made changes to clarify this point.**

7. Related to above, if there truly is a paucity of information, there is no point in performing a systematic review. In that case it may be better to invest time in original research and collecting new data.

**We are aware that many studies have been conducted to investigate prescription practices in primary care in high-income countries, but things are much more blurred in LMICs. Of note, no systematic reviews have been conducted so far to address this research question. We believe that there is a need to put things in order to understand how much research is being done to address such an important issue and identify major gaps that need to be filled. Although we are quite confident that our prevalence estimates regarding antibiotic prescribing are meaningful, we want to highlight the need for better quality research to evaluate patterns of inappropriate use, which is the key problem.**

8. Please clarify why papers published in predatory journals were excluded without looking at the actual quality of the paper. Wouldn't it be possible that a useful high-quality paper would be published in one of these journals, especially if open access fees may be lower than for other journals for authors from LMIC?

**Please refer to our responses to the Editors on this specific issue (point 8 above).**

9. '…as urban any site with a minimum population of 2,000 inhabitants..' This is per what? Per square mile/km? If it is 2000 persons in a very large geographical area, that area shouldn't be classified as urban.

**Thanks for your question. As we mention in the paper, there is no international standard definition for urban/rural areas because every country has its own criteria. In most instances, we could rely on the authors’ definition, but if not explicitly stated we applied the conventional threshold of 2000 inhabitants which refers to a specific settlement without accounting for population density. Of course, this approach has limitations, but we expect negligible impact on our results. In the absence of standardized approaches, this is the best we could do (please see https://blogs.worldbank.org/sustainablecities/what-does-urban-mean).**

10. I have my doubts about the search term 'primary healthcare'. I think this term wouldn't pick up many papers that were performed in primary care/outpatient/community setting. It is also unclear why the authors haven't used MeSH terms as well.

**Thank you for your comment. Please note that our search strategy allowed to capture a wide range of settings and it was broad enough to identify studies that were conducted across multiple tiers of the health systems. As you can see from our PRISMA diagram, the number of initial records exceeds 10,000, and this largely reflects the fact that many studies were conducted in settings other than primary care. However, because the definition of primary care can be heterogeneous across settings, we preferred to stay on the safe side even if that implied a substantial increase in workload during the screening phase. With regards to MeSH terms, please note that we did include a lot; our full search strategies for each database are presented in Supplementary Text S1.**

11. When discussing the reason for limiting to studies preformed >=2010, the authors should also add that in contrast to the effect of interventions which can usually be estimated using studies from different years, meta-analysing something that is almost guaranteed to chance over time, especially given the knowledge we have about trends in antibiotic prescribing, it doesn't make even sense to include relatively old studies in a pooled estimate.

**Thank you for your suggestion. We have included this point in our paper (Lines 188-190).**

12. Please specify missing % for the different variables

**Percentages have been added as suggested.**

13. Summarising inappropriateness proportions across very different conditions, that likely have different 'ideal' prescribing proportions, e.g. see <https://academic.oup.com/jac/article/73/suppl_2/19/4841820>

<https://academic.oup.com/jac/article/73/suppl_2/ii11/4841821>, isn't very informative. Separate estimates should be provided for different conditions.

**We agree that condition-specific proportions would be very useful and informative. Unfortunately, disaggregated data are not available in individual studies, so we cannot provide the information requested.**

14. The study seems to be comparing apples and oranges with 8 studies including only subjects presenting with one pre-specified condition (with likely different ideal prescribing proportions see comment above) and other studies that did not apply any restrictions on reason for seeking care. These studies are not comparable and shouldn't be combined.

**Thanks for raising this issue. Please note that we have conducted subgroup analyses to assess prescribing proportions in studies that included only specific clinical conditions and studies that included all-comers. Forest plots are provided in the supplementary material. We understand your concerns, but you may want to acknowledge that studies including patients with any clinical condition provide an estimate of the average antibiotic prescribing that is still useful and informative. Of course, this should be better interpreted in the light of local epidemiologic patterns as we point out in our discussion. Also please note that reference thresholds, which should be context-specific, are not available.**

15. Why report confidence intervals for study bias?? I don't think confidence intervals make sense to report here, it would be sufficient to just report the percentages without CI.

**Thanks for highlighting this. We fully agree with you that this is inappropriate. We have made changes accordingly.**

16. I don't understand why the author mention a formal comparison of proportion of high-risk studies across different types of countries? Why would this be of interest?

**Sensitivity analyses that restrict to studies with high or low risk of bias are common practice in meta-analyses. Our purpose was to look for differences in antibiotic prescribing proportions that could be due to methodological quality. As discussed in the paper, we could not identify any particular pattern.**

17. '…. And the study population was rarely representative of the target….' The target of the current study, or the target of the original study?

**Here we refer to the target population of the original study, which is one of the aspects to take into account in the assessment of prevalence studies.**

18. Please add a reference for the Hawthorne effect.

**References are now included as suggested.**

19. 'the proportion of prescriptions containing antibiotics varied between 17.8% .. and 79%..' This is so dependent on other drugs and indications, hardly informative at all…

**We understand your concerns. However, as clarified in Lines 308-310 it is important to note that many studies that used prescriptions as denominator reported that all patients received at least one drug (i.e. everyone received a prescription). This not surprising particularly in primary care, where providers often feel “forced” to prescribe or dispense medicines (perhaps just symptomatic drugs like cough suppressants or analgesics) in an attempt to increase patients’ satisfaction and trust. Not giving medicines is often misperceived by patients who look for immediate relief and reassurance. Hence, we believe that those proportions are fully representative of the extent of antibiotic prescribing regardless of other drugs.**

20. 'we could not identify any specific pattern in the distribution of antibiotic rates across levels of country income'. Could be added that this may be due to small sample sizes.

**Thanks for your suggestion. We have included this point in the results section (Lines 301-302).**

21. I2 values are above 98%, again confirming that one may be comparing oranges with apples. Simply using a random effects model and providing prediction intervals doesn't overcome this problem. The sentence 'indicating that a new potential observation in a similar setting would likely yield a proportion of patients receiving antibiotics close to 50%' is therefore also problematic.

**Thanks for your comment. We would like to point out that common methods used to evaluate heterogeneity in meta-analyses seem not to perform very well when the measure being pooled is a prevalence proportion. An I-squared value around 98% is extremely common in meta-analyses of proportions but does not necessarily imply that pooled estimates are totally useless. Also, we did not use conventional meta-analysis method to pool proportion, but rather an approach that is more appropriate when handling prevalence proportions. Importantly, as can be observed in our graphs, about three quarter of the studies included in our final synthesis reported an average proportion of antibiotic prescribing above 40%, and in most cases this proportion ranged between 40 and 60%, thus showing an overall alarming picture in spite of heterogeneity issues.**

22. All antibiotic prescriptions were classified as inappropriate in three studies. This is for the Chinese study likely based on inappropriate methodology to determine inappropriateness. E.g. RTI with a likely viral etiology, can still include a certain proportion of patients that would legitimately receives antibiotics, e.g. see <https://academic.oup.com/jac/article/73/suppl_2/19/4841820> & <https://academic.oup.com/jac/article/73/suppl_2/ii11/4841821>

**Thank you for raising this issue. Please note that the assessment of prescription appropriateness was done by the study authors. We simply reported their judgement, which was based on clinical information. We have better explained this point in Lines 170-171 and Lines 330-332. It is certainly possible that a small proportion of cases were misclassified, but this is unlikely to be significant given that the vast majority of URTIs are self-limiting and do not require an antibiotic treatment.**

23. 'however Watch-group antibiotics accounted for high proportions among Mexico (90.3%). This extremely high percentage doesn't really line up with other studies (on restrictive populations): <https://www.ncbi.nlm.nih.gov/pubmed/30522834>. Of course this is a different population, but I find it at least surprising if use of Watch antibiotics would be more common among less severely ill patients in Mexico. Furthermore, the 90% is also way off the % estimated in another recent study: <https://www.thelancet.com/journals/laninf/article/PIIS1473-3099(18)30547-4/fulltext>

**We agree that the proportion of Watch-group antibiotics is very high in Mexico as reported in our manuscript. However, we would like to stress that this is only one study whereas the reference cited above by the reviewer includes antibiotic consumption among kids nationally. In addition, our study includes only diarrheal condition. We clarify this by indicating that this is just one study and reliability of these estimates is questionable in Lines 396-399.**

24. 'The WHO recommends that the proportion of patients receiving antibiotics in an outpatient setting should be less than 30% [68]. However, this threshold was estabilished somewhat arbitrarily more than two decades ago, due to the lack of evidence on prescription practices and actual needs according to patient's clinical features.' There are examples in UK and EU where condition-specific ideal prescribing proportions were estimated (and compared with real prescribing data):

<https://academic.oup.com/jac/article/73/suppl_2/19/4841820> & <https://academic.oup.com/jac/article/73/suppl_2/ii11/4841821>

<https://qualitysafety.bmj.com/content/20/9/764>

**Thank you for your comment. This is an extremely important point that has been extensively discussed among our team. The lack of reference thresholds makes assessment of prescription practices even more challenging. Estimates from high-resource settings such as Europe or North America are generally inapplicable to LMICs. As we underline in our discussion, the WHO threshold is arbitrary other than outdated, which makes it poorly useful if not potentially misleading. You are definitely right in proposing the use of accurate prescribing data to define ideal prescribing proportions, and this is in line with our suggestion to combine nationally representative AMR surveillance data with GBD data (Lines 429-434). We have also included an additional sentence to better incorporate your considerations (Lines 375-377).**

25. 'A recent published umbrella review'. Please specify that this focused on dental care.

**Specifications were made according to your suggestion. Thank you.**

26. 'or include thorough laboratory testing to match diagnosis with antibiotic use'. This would likely result in unfair comparisons as such testing is typically not available (not even in HIC setting) and in face of uncertainty it would be acceptable that a certain proportion of patients would be treated with antibiotics even if it would not be correct according to strict laboratory tests.

**Thanks for your comments. The limited diagnostic capacity due to unavailability of tests particularly in primary care is by all means an important challenge. However, assessing the appropriateness of antibiotic use without knowing the final diagnosis of patients being evaluated is highly prone to errors. When prescription practices are evaluated using real patients, detailed information regarding their presentation and clinical evolution are key to make an accurate judgement. We have expanded this section further to support our statements (Lines 423-428).**

**Reviewer #2:**

Interesting review, I have a few comments regarding the selection of the studies:

1. The authors said "Conference proceedings and abstracts, commentaries, editorials, reviews, mathematical modelling studies, economic analyses, qualitative studies, and studies published in predatory journals as defined by Beall [13] were excluded": Why is the rationale to exclude Conference proceedings and abstracts? sometimes these studies never get a full manuscript, because the researchers didn't have enough time, so they publish in conference abstract book. Of course, these studies have higher risk of bias and low quality but can provide extra-evidence.  Similar situation are papers from predatory journals (n=5): why not evaluate them and report the quality of them? Sometimes some researchers (specially at LMICs) didn't know the problem regarding predatory journals.

**Thank you for your question. Please see our comment above regarding predatory journals and the reasons for not including such publications in our systematic review. Also note that only 5 papers were excluded for this reason, thus likely minimally affecting our findings and conclusions. As for conference abstracts, one of the biggest issues that we often encounter besides quality concerns is that abstracts do not contain sufficient information to make at least basic judgements and proceed with analyses. The decision to not include abstracts is controversial, and there are good reasons both pro and against. At some point, we also need to consider feasibility aspects as systematic reviews always require a huge effort in terms of study screening and selection in order to ensure the highest methodological rigour.**

2. Figure 2:  I'm a bit surprise about the low risk of "Reliability & validity of method used for measuring prescriptions" and "avoidance of inappropriate exclusions", because one of the most frequent data source were medical records and, in general, the quality from these records is not too high.  I suggest to discuss a bit about it.

**Thanks for bringing up this point. Medical records and drug prescription audits, that were used in the largest majority of studies, are good data sources to estimate the proportion of antibiotic prescriptions. For this reason, we considered these sources as low risk. However, such sources are generally unsuitable to accurately evaluate the appropriateness of prescription, which is the biggest limitation that we have highlighted in our review. We have explained this concept more in detail as suggested (Lines 287-291).**

3. Figure 3:  I suggest to don´t present an overall OR, at least you can present in sub-groups based in the type of denominator.  I suggest only present as was presented in the supplementary material, because the study population is different

**Thank you for this comment. The reason for presenting the forest plot with all studies was related to the finding that in studies that used the total number of patients as denominator, all patients actually received drug prescriptions. Therefore, we thought that combining studies that used different denominators could be acceptable. However, we agree with you that the study populations differ, so we have modified Figure 3 in order to show two strata defined by denominator.**

4. Meta-regression and heterogeneity: I suggest to evaluate the studies used for meta-regression, maybe only use studies with similar denominator

**Thanks for your suggestion. We did run several meta-regression models and found similar results, so we decided to present only selected findings as an example. Actually, this further strengthens the idea that methodological limitations and poor reporting observed across several studies represent a huge challenge when trying to summarize the evidence on prescription practices.**

**Reviewer #3:**

This is an important study that has the potential to contribute in informing policy and practice in tackling antimicrobial resistance as it attempted to analyse prescription practice in LMICs. It is properly conducted and very well written.  The dearth and poor quality of the studies in these settings as demonstrated by the review limits the drawing of any strong conclusion as stated by the authors on the practice and appropriateness of antibiotic prescriptions. More importantly, the interpretation of such results should be carefully crafted and contextualized as every year inadequate access to antibiotics kills nearly 6 million people, including a million children who die of preventable sepsis and pneumonia mostly in LMICs. Therefore, interpretation of the data requires caution in the face of this reality

**Thank you very much for your comments and considerations. We agree that it is very difficult to draw strong conclusions and as suggested by editorial team we toned down our conclusion (Lines xxx). Our work highlights the need for better approaches to investigate prescription practices. Such methodological limitations apply to any context and become extremely relevant when attempting to assess appropriateness of prescription.**

Major comments:

1. The operational definition of primary care as given by the authors is the foundation of the review. However, the definition is bundled into non-specific categorisation that its application limits ultimate utility of the study for any meaningful policy and practice implications. The level of tier of the health system (e.g. referral/tertiary/provincial/district hospital; health center/polyclinic/health station/clinic) in a country determine and regulate the system that determine prescription practice (e.g. the cadre of health worker;  type and class  of medicines/antibiotics should be available and used). Therefore, presenting the data by specific health facility would help to inform any potential antimicrobial/antibiotic stewardship programmes in LMICs and enhance the relevance and utility of the study.

**Thank you for raising this issue. We agree with you that stratifying by type of facility would be interesting and certainly useful. However, this is not feasible owing to the substantial heterogeneity in terms of health system structure and definitions used across settings, but also due to the limited details provided in many studies. We recognize that this is an important limitation, which further points to the urgent need for more accurate reporting in research on this issue. We have acknowledged this in our discussion (Lines 364-367).**

2. The WHO AWaRe classification of antibiotics as the authors mentioned is to assist antibiotic stewardship efforts and promote optimal use so as to prevent the development of drug resistance. The classification underline that the narrow-spectrum antibiotics in the Access group are the preferred treatment option for most infections (e.g Respiratory tract) and are  also thought to have a lower ecologic impact regarding the selection and spread of antibiotic resistance than broader-spectrum agents. Therefore, Access group antibiotics should therefore constitute the majority of antibiotic use in the outpatient setting and overall. As part of the outcome measurement  of the current Global Programme of Work of WHO, countries should strive to ensure that Access group antibiotics constitute more than 60% of the overall antibiotic use. Authors should frame their analysis, presentation and interpretation likewise.

**Thank you for your suggestion. We expanded the paragraph on AWaRe classification in the Discussion section (Lines 389-401). However, we could only analyse 16 studies out of 48 which included individual antibiotic information. There is only one study from each country which limits our overall interpretation as WHO recommends having at least 60% Access of total antibiotic use at the country level. We added considerations on these issues.**

3. While recognising the paper will undergo statistical review, I wonder on the value of the pooled estimates.

**Thank you for this comment. We understand your concerns and we are well aware that pooled estimates need to be interpreted with great caution in the presence of high levels of between-study heterogeneity. Yet, it should be acknowledged that common methods used to evaluate heterogeneity in meta-analyses do not seem to perform very well when the measure being pooled is a prevalence proportion. An I-squared value around 98% is extremely common in meta-analyses of proportions but does not necessarily imply that the pooled estimate is totally useless. Importantly, as can be observed in our graphs, about three quarter of the studies included in our final synthesis reported an average proportion of antibiotic prescribing above 40%, and in most cases this proportion ranged between 40 and 60%, thus showing an overall alarming picture in spite of heterogeneity issues. We have toned down the conclusions as also suggested by the editors.**

Minor comments:

1. Authors' use of 'community' level in the paper has to be clarified or removed.

**Thanks for your suggestion. We have removed that term.**

2. Clarify and correct use of antimicrobial vs antibiotic in the text

**Thanks for raising this issue. We have consistently used the term “antibiotic” to refer to any anti-bacterial drug in line with current standards. We purposely avoided the term “antimicrobial” which includes any anti-infective agent (e.g. anti-viral, anti-mycotic, anti-bacterial, anti-parasitic) because only anti-bacterial drugs were examined across included studies.**

3. Ref 68 is obsolete and need to be removed

**Thanks for your suggestion. Although we agree with the reviewer that this is an old reference (now ref. 78), we decided to keep it because it is the source of the information regarding the recommended threshold for the proportion of antibiotic prescriptions in outpatient care according to WHO. As we point out in the manuscript, this threshold is arbitrary and may not apply to a variety of settings.**

**Reviewer #4:**

I confine my remarks to statistical aspects of this paper. These were well done and I recommend publication.

**Thank you very much.**
